# Editorial: Brain-body interactions underlying comorbid depression and other mood disorders

**DOI:** 10.3389/fpsyt.2026.1754218

**Published:** 2026-01-19

**Authors:** Mounira Banasr, Kenneth E. McCarson, Daniel T. Christian, Vanja Duric

**Affiliations:** 1 Campbell Family Mental Health Research Institute of CAMH, Toronto, ON, Canada; 2 Department of Psychiatry, University of Toronto, Toronto, ON, Canada; 3 Department of Pharmacology and Toxicology, University of Toronto, Toronto, ON, Canada; 4 Department of Pharmacology, Toxicology and Therapeutics, University of Kansas Medical Center, Kansas, KS, United States; 5 Department of Physiology and Pharmacology, Des Moines University Medicine and Health Sciences, Des Moines, IA, United States

**Keywords:** biomarkers, brain-body crosstalk, depression, diagnosis, systemic illness, treatment

Depression is among the most prevalent psychiatric disorders globally with a disabling impact on individual psychological well-being and overall health (1). Beyond its psychological symptoms, depression is increasingly recognized as a systemic illness with complex interactions between the brain and peripheral organ systems (2). Chronic stress plays a pivotal role in these interactions, exerting profound effects on the central nervous system (CNS) and disrupting brain’s regulatory influence over physiological processes. This dysregulation contributes to the onset and progression of psychiatric and systemic illnesses, including cardiovascular disease, diabetes, chronic pain, and neurodegenerative disorders (3).

The mechanisms underlying the brain–body interactions remain incompletely understood, but growing evidence points to neuroendocrine, immune, and inflammatory pathways as key drivers (4). Stress can compromise the integrity of the blood–brain barrier, enabling peripheral inflammatory signals to affect brain function and behavior (5, 6). This interplay contributes to the development of mood disorders but also complicates the management and treatment of both depression and systemic illnesses. Neuroendocrine dysregulation, immune activation, and inflammatory cascades collectively impair brain’s control over peripheral systems, allowing inflammatory mediators to penetrate the CNS and alter neural functions. Understanding these mechanisms is critical for developing integrated treatment strategies that address both mental and physical health outcomes.

The studies featured in this Research Topic add complementary evidence that depression is not an isolated disorder, but a multidimensional illness shaped by genetic, metabolic, and immunological factors. They highlight 1) potentially novel biomarkers such as lipid ratios and inflammatory proteins, 2) important comorbidities spanning chronic diseases and neurodegenerative conditions, and 3) innovative therapeutic interventions including digital health technologies. Collectively, these findings advance our understanding of depression as a systemic illness requiring holistic approaches that bridge neuroscience, immunology, endocrinology, and emerging digital therapeutics. This perspective accentuates the need for interdisciplinary research and clinical models that move beyond symptom management towards strategies designed to improve outcomes and quality of life for individuals affected by depression and its comorbidities.

## Biomarkers and physiological correlates of depression

Compared to neurodegenerative illnesses, cancer or cardiovascular disorders, finding biomarkers for depression has been challenging despite the need of measurable indicators for improving either diagnosis, prognosis, or treatment efficacy. Several papers in this Research Topic raise the possibility that biomarkers of depression could be found when considering other systems than the brain and underscore the role of systemic inflammation, lipid metabolism, and neurophysiological dysfunction in depressive disorders. Li et al. identify the Platelet/High-Density Lipoprotein Cholesterol Ratio (PHR) as a potential biomarker for depression among individuals with chronic opioid use. Their analysis of the National Health and Nutrition Examination Survey (NHANES; 2007–2018) data reveals that elevated PHR is independently associated with depression, even after adjusting for demographic and clinical covariates. They also suggest that beyond a certain threshold, PHR sharply increases depression risk. Subgroup analyses indicate stronger associations in younger individuals and those with obesity, pointing to systemic inflammation and lipid dysregulation as key contributors. These findings emphasize the need to incorporate metabolic and inflammatory markers into mental health assessments especially in depressed individuals with chronic opioid use.

Furthermore, Jiang et al. investigated the Framingham Steatosis Index (FSI), a diagnostic indicator of accumulation of fat in liver, and its potential association with depression. Using data from nearly 20,000 participants, they demonstrate that higher FSI values correlate with increased depression risk. This study highlights a role of metabolic dysfunction as well as hormonal imbalances, and inflammation in depression pathophysiology. The authors suggest that screening for fatty liver indicators and related metabolic disturbances could improve preventive strategies for depression.

Sleep disturbances and cognitive impairments are hallmarks of depression. Fang et al. showed a potential link between sleep efficiency, event-related potentials (ERPs), and levels of serum C-reactive protein (CRP) in patients with depression. Their results indicate that poor sleep efficiency is associated with prolonged ERP latencies and high levels of CRP and suggest that chronic inflammation can mediate cognitive dysfunction in depression. This study fuels the literature suggesting that addressing sleep quality and inflammatory status is important when considering therapeutic interventions for depression.

Genetic and immunological factors also contribute to the depression pathology and its comorbidities. Liu et al. report shared biomarkers—CD163 and KLRB1—between depression and diabetic kidney disease, suggesting involvement of common inflammatory and immune pathways as risk factors. Their comprehensive analysis, including linkage disequilibrium score regression and gene expression profiling, identifies 83 crosstalk genes and eight hub genes involved in immune regulation. These findings further reinforce the systemic nature of depression and open avenues for targeted therapies addressing shared mechanisms across comorbid conditions. Finally, Zhang et al. provide evidence of neuroinflammation and brain function abnormalities in HIV-associated depressive disorders. Using resting-state fMRI and peripheral immune profiling, they demonstrate altered connectivity patterns and elevated inflammatory markers in HIV-positive individuals with depression, underscoring the detrimental effects of immune dysregulation on the brain function.

Collectively, these studies position potential biomarkers—ranging from lipid ratios and liver indices to inflammatory proteins and genetic signatures—as critical tools for advancing precision psychiatry. They also emphasize the interplay between peripheral and central processes in stratifying depressive phenotypes.

## Depression in specific populations and comorbid conditions

Depression manifests differently across populations and comorbidities, highlighting the heterogeneity of the disorder and the need for tailored interventions. Several studies in this Research Topic showcase these differences and nuances. As mentioned above, Li et al. highlight the role of lipid metabolism and systemic inflammation as a depression risk in depressed individuals with chronic opioid use. These findings call for integrated treatment approaches that encompass substance use disorder, depression, and metabolic health. In depressed patients with Parkinson’s disease, Xu et al. reveal a bidirectional relationship between depression and activities of daily living (ADL) that are mediated by cognitive function. Their longitudinal analysis shows that depression predicts functional decline, while impaired ADL exacerbates depressive symptoms. In PD-associated depression, decline in cognitive function is a key mediator, suggesting that interventions aimed at enhancing cognition could improve quality of life for PD patients.

Moreover, Zhang et al. investigated HIV-positive individuals as another vulnerable group. The authors report high rates of depression among men who have sex with men living with HIV, linking these symptoms to aberrant immune responses and brain connectivity alterations. These findings emphasize the need for mental health services that are integrated into HIV care, addressing both neuropsychiatric and immunological dimensions. Interestingly, Chen et al. uncover a genetic overlap between psychiatric disorders—including depression, bipolar disorder, and schizophrenia—and hemorrhoidal disease. Their genome-wide cross-trait analysis identifies pleiotropic genes and causal relationships, suggesting shared biological mechanisms. While the clinical implications of this association remain to be fully elucidated, these findings expand our understanding of depression as a systemic disorder with unexpected genetic links.

These studies collectively reinforce the concept that depression is deeply intertwined with physical health, genetic predispositions, and social determinants. Personalized treatment plans that consider these factors are essential for improving outcomes across diverse patient populations.

## Innovative therapeutic interventions

Advancements in therapeutic technologies are creating new opportunities for managing depression and its primary comorbidities, such as anxiety. Premkumar et al. contributed a study to this Research Topic focusing on evaluating the effect of self-guided Virtual Reality Exposure Therapy (VRET) on social anxiety. The authors conducted a randomized controlled trial demonstrating that VRET significantly reduces public speaking anxiety and physiological arousal, especially when augmented with biofeedback such as heart rate and frontal alpha asymmetry monitoring. These benefits were sustained at one-month follow-up, indicating that immediate biofeedback enhances VRET by empowering individuals to regulate anxiety responses in real time, potentially allowing them to develop adaptive coping strategies. The emergence of innovative therapies such as VRET with biofeedback offers hope for more effective and personalized treatment strategies, in particular when systemic parameters are included.

## Conclusion

The studies featured in this Research Topic collectively accentuate and support the multifaceted nature of depression. From biomarkers and genetic predispositions to systemic inflammation and neurophysiological dysfunction, the evidence points to a complex web of brain–body interactions that shape the onset, progression and the severity of depressive disorders. The high prevalence of comorbidities—spanning chronic pain, diabetes, HIV, Parkinson’s disease, and less commonly considered conditions such as hemorrhoidal disease—further highlights the urgent need for integrated, interdisciplinary approaches to psychiatric research and treatments. Digital therapeutics, including VR-based interventions, represent a shift toward delivering personalized, technology-driven mental health care, offering scalable and accessible solutions for anxiety and depression. With continued evolution of technology, incorporating these tools into mainstream psychiatric care could revolutionize treatment paradigms, making mental health support more inclusive and effective. As we continue to unravel the biological and systemic underpinnings of depression, future research must prioritize translational approaches that bridge neuroscience, immunology, endocrinology, and technology for disorder monitoring and therapeutics. This editorial highlights the need for sustained collaboration and innovation to advance the field of psychiatric research. By embracing a holistic view of depression that encompasses both brain and body, we can move toward more comprehensive care models that improve outcomes and quality of life for individuals affected by depression and its various comorbidities.
